# Hodgkin's Lymphoma Revealed by Hemophagocytic Lymphohistiocytosis in a Child

**DOI:** 10.1155/2014/851392

**Published:** 2014-09-29

**Authors:** Sarra Benmiloud, Mohamed Hbibi, Sana Chaouki, Sana Abourazzak, Moustapha Hida

**Affiliations:** ^1^Unit of Pediatric Hematology-Oncology, Department of Pediatrics, University Hospital Hassan II, Faculty of Medicine and Pharmacy, University of Sidi Mohamed Ben Abdellah, 30000 Fez, Morocco; ^2^Unit of Pediatric Hematology-Oncology, Department of Pediatrics, Mother-Child Hospital, University Hospital Hassan II, Faculty of Medicine and Pharmacy, University of Sidi Mohamed Ben Abdellah, BP 1893, Km 2.200, Sidi Hrazem Road, 30000 Fez, Morocco

## Abstract

Hemophagocytic lymphohistiocytosis (HLH) is a severe life-threatening disorder, responsible for extensive phagocytosis of hematopoietic cells and causing a multisystem organ failure. If lymphomas are common causes of HLH, the association with Hodgkin's lymphoma is rarely described in children. We report a case of a 9-year-old boy presenting with HLH as an initial manifestation of Hodgkin's lymphoma. He has been suffering from persistent high fever, asthenia, weight loss, and hepatosplenomegaly with no lymphadenopathy. The diagnosis of HLH secondary to infectious disease was initially worn. The patient received high-dose intravenous immunoglobulin with broad-spectrum antibiotics. However, his state got worse with the onset of dry cough and pleural effusion. Histopathologic examination of pleural fluid showed the presence of Reed-Sternberg cells. The outcome was favorable after treatment by corticosteroid and chemotherapy. Hodgkin's lymphoma revealed by HLH is a source of delayed diagnosis and should be borne in mind in children.

## 1. Introduction

Hemophagocytic lymphohistiocytosis (HLH) is a severe life-threatening disorder causing a multisystem organ failure. It is characterized by an excessive and uncontrolled immune response, due to cytokine dysregulation and lymphohistiocytic proliferation [1, 2]. The HLH is usually a secondary reaction to infection, medication, autoimmune, or neoplastic diseases. Hematologic malignancies are a well-known HLH etiology, but the combination of Hodgkin's lymphoma (HL) and HLH in the pediatric population is rarely reported at the time of diagnosis.

We report a novel pediatric case of HL revealed by HLH as an initial manifestation, illustrating the diagnostic difficulties and the interest of rapid treatment.

## 2. Case Report

A 9-year-old boy, with no past medical history, was admitted for a persistent high fever that reached 40°C, evolving for one month, associated with anorexia, asthenia, vomiting, and significant weight loss. Clinical examination found the child in poor physical condition, febrile to 39.5°C, and pale. Abdominal palpation found a hepatosplenomegaly. The rest of the physical examination demonstrated lenticular cervical and inguinal lymph nodes.

**Figure 1:**
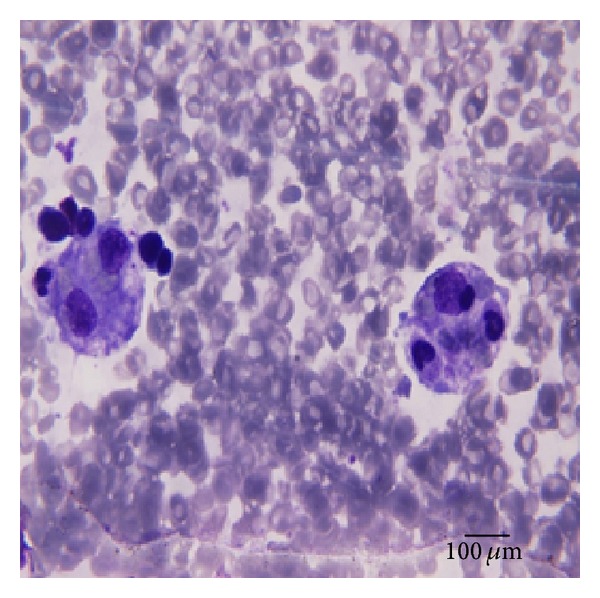
Image showing two macrophages with hemophagocytosis in bone marrow aspiration smear (magnification ×1000).

Laboratory tests revealed leukopenia (white blood count = 1080/mm^3^, normal: 4000–10000/mm^3^), neutropenia (neutrophil = 750/mm^3^, normal: 3000–5600/mm^3^), lymphopenia (lymphocytes = 120/mm^3^, normal: 3000–5500/mm^3^), anemia (hemoglobin = 6.6 g/dL, normal: 11.5–14.5 g/dL), thrombocytopenia (platelet count = 79000/mm^3^, normal: 150000–450000/mm^3^), elevated C-reactive protein (171 mg/L, normal: 0–6 mg/L), abnormal liver function (serum glutamate oxaloacetic transaminase = 258 IU/L, normal: 5–34 IU/L; serum glutamate pyruvate transaminase = 168 IU/L, normal: 0–55 IU/L), elevated lactate dehydrogenase (973 IU/L, normal: 0–248 IU/L), hyperferritinemia (703 *μ*g/L, normal: 20–250 *μ*g/L), hypertriglyceridemia (3.7 g/L, normal: 0–1.50 g/L), normal fibrinogenemia (3.9 g/L, normal: 2–4 g/L), and hypoalbuminemia (19.2 g/L, normal: 33–50 g/L). The bone marrow aspiration showed the presence of numerous macrophages with hemophagocytosis without evidence of malignancy (Figure 1).

The diagnosis of HLH was worn based on six of eight diagnostic criteria: fever, hepatosplenomegaly, pancytopenia, hyperferritinemia, hypertriglyceridemia, and bone marrow hemophagocytosis [1]. The bacteriological (blood, urine and stool cultures, tuberculosis tests, and mycoplasma serology), viral (serology of Epstein-Barr virus (EBV), cytomegalovirus, parvovirus B19, human immunodeficiency virus, and hepatitis A, B, and C), and parasite (toxoplasmosis, leishmaniasis) evaluations were negative. Immune tests (antinuclear antibodies and anti-DNA and rheumatoid factor) were normal. The search for EBV by polymerase chain reaction (PCR) could not be performed due to the lack of financial patient's resources.

**Figure 2:**
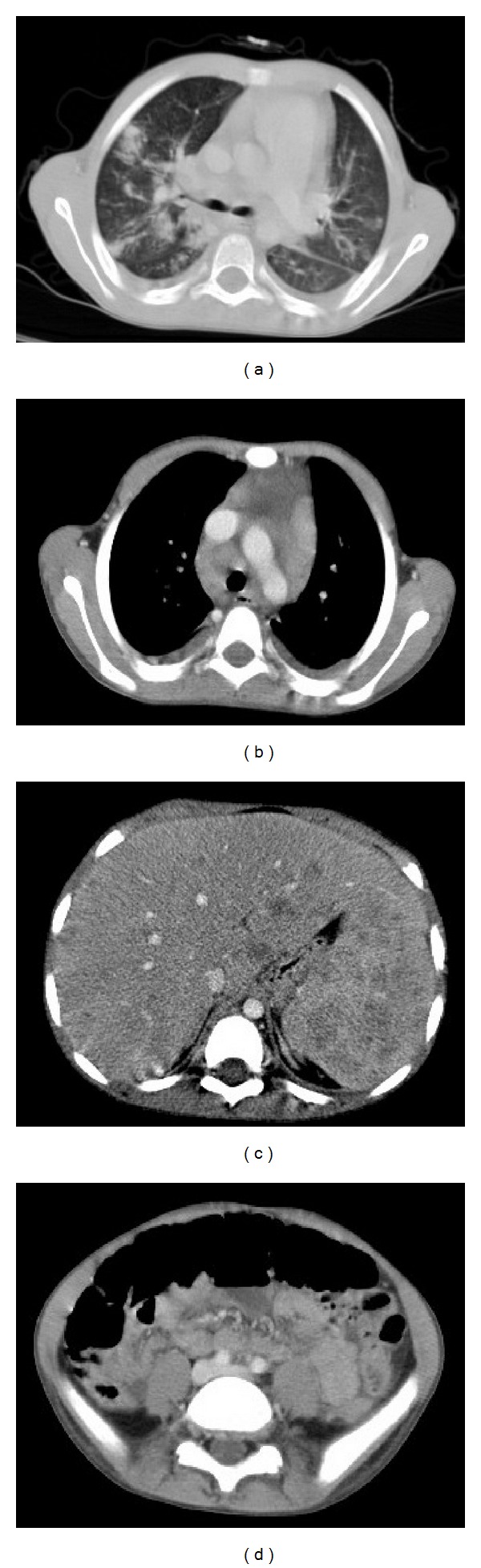
Thoracoabdominopelvic computed tomography in axial sections demonstrating scattered nodules at different parenchymal lung segments, a right parenchymal lung condensation, and minimal pleural effusion (a), multiple mediastinal enlarged lymph nodes (b), hepatosplenomegaly containing multiple nodular hypodense lesions (c), and hilar and para-aortic infracentimetric lymph nodes (d).

**Figure 3:**
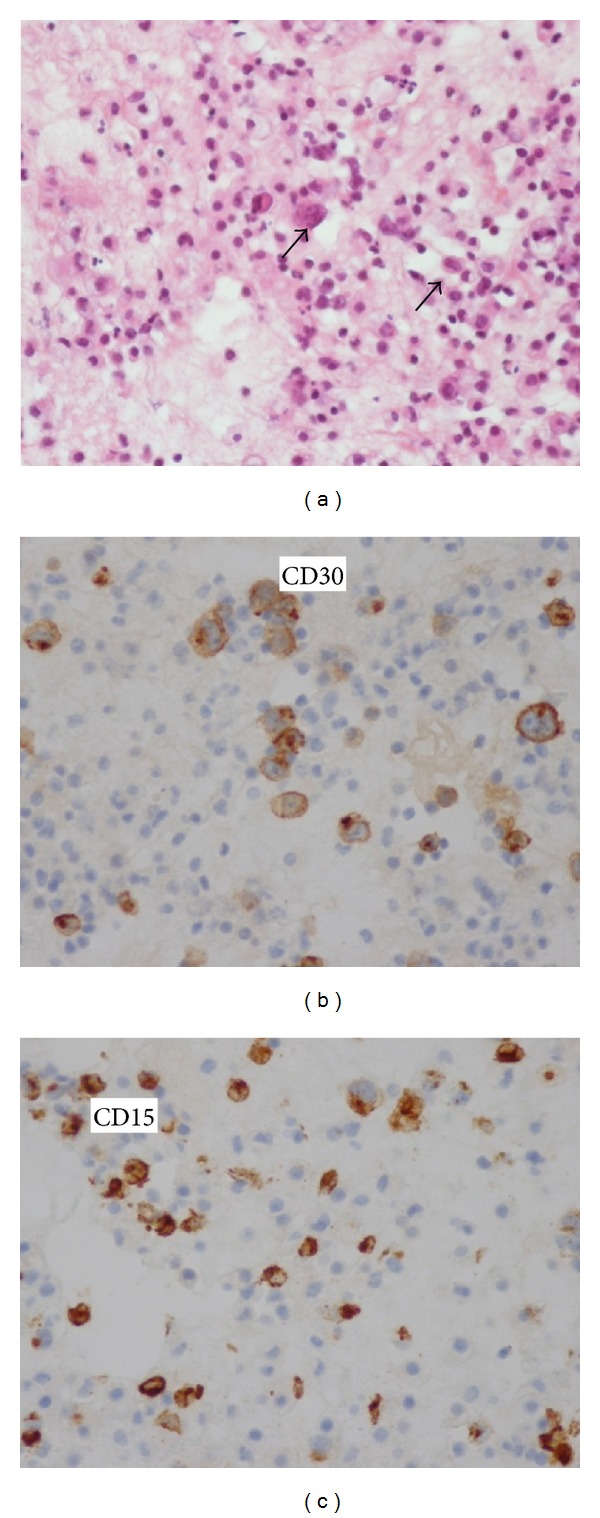
Histopathological examination of pleural fluid: cytological spreading showing atypical cells with cusped nuclei (arrows) on a background rich in neutrophils (magnification ×250) (a), immunocytochemistry expression of CD 30, and CD15 by tumor cells (b and c).

The diagnosis of HLH secondary to infectious disease was initially suspected. The patient received broad-spectrum antibiotics and high-dose intravenous immunoglobulin. However, his condition had worsened with the onset of dry cough and dyspnea. The chest X-ray demonstrated an alveolar syndrome in the lower lobe of the right lung and a bilateral minimal pleural effusion. The thoracoabdominopelvic computed tomography revealed the presence of scattered nodules at different parenchymal lung segments, measuring 15 mm for the largest diameter, a right parenchymal lung condensation, multiple mediastinal enlarged lymph nodes measuring 14 mm for the largest, minimal pleural and pericardial effusions, hepatosplenomegaly containing multiple millimetric nodular hypodense lesions, and hilar and para-aortic infracentimetric lymph nodes (Figure 2). Echocardiography objectified minimal pericardial effusion with normal cardiac function. Bacteriological examination of pleural fluid was negative. Histopathological examination of pleural fluid revealed the presence of Reed-Sternberg cells that was positive for the anti-CD15 and anti-CD30 antibodies (Figure 3); some of these cells were positive for the anti-CD20 antibody. The anti-CD3 antibody was positive in the reactive lymphocytes. EBV-encoded RNA (EBER) in situ hybridization for detecting and localizing latent EBV in patient's HL cells was not done, because this technique is not available in our country's laboratory. Bone marrow biopsy showed no bone marrow infiltration by tumor cells. Due to a drop of patient's performance status, a biopsy of mediastinal lymph nodes or pulmonary lesions could not be made.

The diagnosis of a HL associated with a HLH was worn based on the pathological study of pleural fluid and imaging data. However, no genetic mutation could be studied due to the lack of financial patient's resources. The patient received initially three bolus of methylprednisolone, followed by chemotherapy combining two monthly courses of OPPA (vincristine, doxorubicin, procarbazine, and prednisone) and four monthly cycles of COPP (cyclophosphamide, vincristine, procarbazine, and prednisone). The evolution was slowly favorable. After 6 weeks, the pleural effusion and the hepatosplenomegaly disappeared; the laboratory tests were normal. Currently we are at 20-month follow-up; the child is asymptomatic with no residual disease or relapse.

## 3. Discussion

HLH is an excessive and uncontrolled immune response producing large quantities of inflammatory cytokines. It can be primary, related to several molecular defects (mutations in perforin 1, UNC 13D, syntaxin 11, syntaxin-binding protein-2, SH2D1A, RAB27A, and XIAP or a defect on chromosome 9q21.3-22), or secondary to infections, autoimmune diseases, chronic inflammatory disorders, acquired immunodeficiencies, and various malignancies, mainly hematological malignancies [1, 2]. Clinically, there is a persistent high fever associated with hepatosplenomegaly. Biologically, the diagnosis is suggested by the association of bicytopenia with hyperferritinemia, altered liver function, hypertriglyceridemia or hypofibrinogenemia, increased soluble CD25 levels, and low or absence of natural killer (NK) cells activity. Histologically, there is a significant hemophagocytosis in bone marrow, spleen, or lymph nodes [1]. The identification of the causal disease in the secondary forms is mandatory because only its treatment will stop HLH.

HLH occurring during malignancies has been reported previously in the literature, most commonly in the case of leukemia and lymphomas (more often T or NK phenotype) especially in adult patients [3–5]. It is probably the secretion of proinflammatory cytokines (interferon-*γ*, tumor necrosis factor *α*, interleukin-2 (IL-2), IL6, IL-8, IL-10, IL-12, IL-18, and IL-4), by malignant cells, which contribute to this immune dysregulation. In T or NK lymphomas, it seems that the uncontrolled production of cytokines is caused by EBV [3]. Studies in children, who developed HLH in the sitting of acute lymphoblastic leukemia, suggested that the chemotherapy combined with malignancy predisposes to defects in T-cell and NK-cell function and also to infections which triggered HLH [6]. Sometimes, HLH was reported as the first presentation of malignancies, as in our case, probably due to the release of cytokines induced by activated macrophages and T cells [4, 6, 7].

Among HLH secondary to lymphoma, HL is often described in children [6–9]. Even though EBV is significantly associated with HL, the association of HLH and HL is uncommon at the time of diagnosis [7–9]. It seems that HLH could have a role in determining the clinical manifestations of the primary disease, from silent clinical forms to fulminant evolutions, which often lead to delay in diagnosis as in our case [3]. The largest study of patients with HL and HLH shows that the main features of this association are male predominance, disseminated disease, a high proportion of lymphocyte depletion and mixed cellularity histological subtypes, and a strong association with EBV which was detected in the tumor cells of 94% of cases that suggest a major pathogenic role of EBV in this association [8, 10].

When HLH is diagnosed, the search for a trigger is imperative for the prognosis. In our observation, diagnostic difficulties were due to the rarity of the association of HL and HLH in children at the time of diagnosis and the major alteration of physical condition which made delicate explorations. The diagnosis is easy when the HL manifests as diffuse adenomegalies accessible for biopsy. However, in case of atypical manifestations, histological studies with biopsies are required. Studies in patients with malignancy reported that the presence of HLH is a negative prognosis factor with a high mortality rate, imposing an early and appropriate therapy [3, 6]. According to the literatures, the prognosis of patients in the case of association of HL and HLH is extremely poor [8, 10]. Despite therapy, survival is short. But if the treatment is successful, the survival rate comes back as expected [3].

## 4. Conclusion

HLH as an initial presentation of HL is rare in children. This association is a source of delayed diagnosis and should be borne in mind, especially as the prognosis depends on the rapidity of the treatment including early initiation of chemotherapy.
